# Clinical characteristics and prognosis of primary pancreatic carcinoid tumors: A report of 13 cases from a single institution

**DOI:** 10.3892/ol.2014.2776

**Published:** 2014-12-08

**Authors:** FENG-HUA LIU, CHONG WANG, YA-LING XING, JIANG-HUA WU, YONG TANG

**Affiliations:** 1 Department of Gynecological Cancer, Key Laboratory of Cancer Prevention and Therapy, Tianjin Medical University Cancer Institute and Hospital, Tianjin 300060, P.R. China; 2 Department of General Surgery, Tianjin First Central Hospital, Tianjin 300190, P.R. China; 3 Department of Pancreatic Cancer, Key Laboratory of Cancer Prevention and Therapy, Tianjin Medical University Cancer Institute and Hospital, Tianjin 300060, P.R. China; 4 Department of Pathology, Key Laboratory of Cancer Prevention and Therapy, Tianjin Medical University Cancer Institute and Hospital, Tianjin 300060, P.R. China

**Keywords:** pancreatic carcinoid tumor, diagnosis, treatment, prognosis, pathology

## Abstract

The present study aimed to analyze the diagnosis and treatment of 13 cases of pancreatic carcinoid tumors during a 56-year period at the Tianjin Medical University Cancer Institute and Hospital (Tianjin, China). The data from 13 cases, consisting of 5 males and 8 females (mean age, 50 years), were collected and analyzed. Hematoxylin-eosin and immunohistochemistry staining were performed to investigate the expression of neuron-specific enolase (NSE), cytokeratin (CK), chromogranin A (CgA) and synaptophysin (Syn) in the tumors. The affected patients suffered abdominal and/or back pain without typical carcinoid syndrome. Radical resection was performed in 10 cases and regional resection in one case. The remaining two patients exhbited remote metastasis, and so were treated with single and double bypass surgery (choledochojejunostomy and gastrojejunostomy/choledochojejunostomy, respectively). The expression of CK, Syn, CgA and NSE was positive in nine (69.23%), 10 (76.92%), five (38.46%) and eight (61.54%) cases, respectively. The median survival time was 26.6 months. In conclusion, in patients with pancreatic carcinoid tumors that exhibit no typical carcinoid syndrome, such as those in the present study, the diagnosis can be confirmed by pathological examination and surgery is the most effective treatment.

## Introduction

Pancreatic carcinoid tumors are rare malignancies. Modlin *et al* (1) reported that pancreatic carcinoid tumors account for 0.55% of all carcinoid tumors. However, there is no globally accepted method for the diagnosis and treatment of pancreatic cancer. In the present study, the clinical data of 13 hospitalized patients is reviewed, and the diagnostic and therapeutic methods are discussed.

## Materials and methods

Between January 1954 and May 2010, 13 patients with pancreatic carcinoid tumors were treated in Tianjin Medical University Cancer Institute and Hospital (Tianjin, China). The clinical data of these patients were collected and analyzed. Hematoxylin-eosin and immunohistochemistry (IHC) staining of the 13 patient tissues were performed. The expression of neuron-specific enolase (NSE), cytokeratin (CK), chromogranin A (CgA) and synaptophysin (Syn) were observed by IHC staining. In these experiments, 10% of cancer cells were stained brown. Staining intensity was scored according to the percentage of positively stained cells as follows: −, <5%: +, ≥5 but <25%; ++, 25–50%; and +++, >50%. The clinical data of the patients was collected by telephone or during outpatient service follow-up during the post-operative period until August 2011. Consent was obtained from the patient’s families.

### Statistical analysis

The patient survival curve was generated by Kaplan-Meier analysis. SPSS 13.0 was used for the statistical analysis of the data (SPSS, Inc., Chicago, IL, USA).

## Results

### Patient characteristics

Between 1954 and 2010, 414 cases of carcinoid tumors were admitted to Tianjin Medical University Cancer Institute and Hospital and 13 patients (3.14%, 13/414), 5 male and 8 female, were diagnosed with pancreatic carcinoid tumors. The mean age was 50 years (range, 39–64 years). Details of the patient information are presented in Table I.

### Clinical presentation

Emaciation and jaundice were observed in one case, and one patient had headache symptoms. In addition, three patients presented with pain in the back. Epigastric pain was exhibited by five cases, from which, two patients presented with emaciation and nausea simultaneously; the remaining three patients were diagnosed with pancreatic carcinoid tumors during physical examination. None of the patients exhibited signs of carcinoid syndrome.

### Laboratory examinations

One case presented with a slight increase in carcinoembryonic antigen (CEA; 5.25 μg/l; normal range, 0–5 μg/l) and another case exhibited characteristics of an elevated level of ferritin (240.8 μg/l; normal range, 13–150 μg/l). None of the patients had abnormal levels of gastrointestinal tumor markers [carbohydrate antigen (CA)199, CEA and CA724], and an increased 5-Serotonin (5-HT) content in the serum and an elevated 5-hydroxyindoleacetic acid (5-HIAA) level in the urine were not detected prior to the pathological diagnosis.

### Tumor size, location, and metastasis

**Figure 1:**
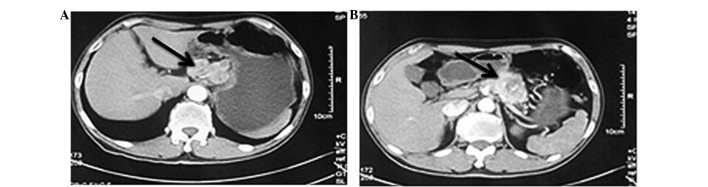
Computed tomography images from patient no. 12, a 60-year-old with a two-month history of abdominal pain, showing masses located on the pancreatic body and tail, with lower density and irregular shadows. (A) The venous phase showing the main pancreatic duct expansion, and (B) the delayed phase with slightly uneven density, showing a patchy low-density area of necrosis; (arrows indicate the location of the tumor).

Pancreatic carcinoid tumors were detected in two cases during the physical examination, without any symptoms, and the remaining patients exhibited large-sized tumors, with a mean diameter of 7.1 cm, as shown in Fig. 1. Tumors were found on the head of the pancreas in eight cases and on the body and tail in the remaining five cases. Two patients presented with hepatic metastasis, one with celiac metastasis and one with spleen metastasis. A portal tumor thrombus was observed in two patients.

### Imaging features

Using B-mode ultrasonic detection, it was found that the solid tumors had hypoechoic, irregular margins, non-uniform echo in the tumor, no significant blood flow and no dilated pancreatic ducts. The initial diagnostic report of the computed tomography (CT) and magnetic resonance imaging scanning images provided evidences for pancreatic neuroendocrine or islet-cell tumors. The majority of the CT images showed heterogeneous enhancement of the arterial phase as a characteristic of the neuroendocrine tumor, which could be differentiated into pancreatic ductal adenocarcinoma.

### Therapy methods

In total, 10 cases were treated with radical resection. Pancreatoduodenectomy was performed on 4 patients, 5 patients were treated with distal pancreatectomy, and local resection was performed on the remaining patient for the small tumor (1 cm^3^). For the two patients with portal tumor thrombi, as described previously, a pancreatoduodenectomy was performed, and the embolus was removed at the same time. However, radical surgery could not be performed on two patients due to the remote metastasis and they were therefore treated with cholangiojejunostomy and gastrojejunostomy/choledochojejunostomy, respectively. Biopsy and administration of somatostatin were performed simultaneously. Four patients received somatostatin at a dosage of 0.9 mg/day, the durations varied between patients.

### Pathology characteristics

**Figure 2:**
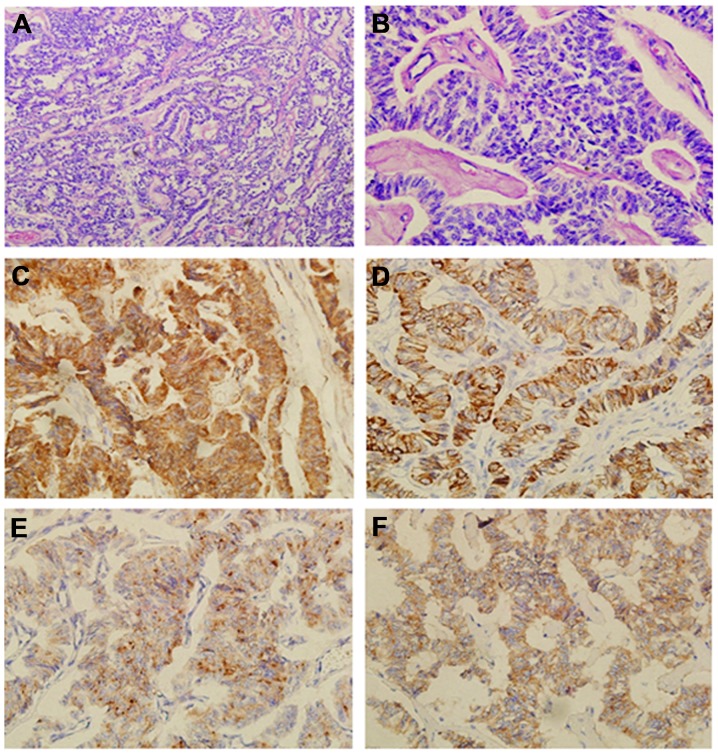
Hematoxylin and eosin (HE) and specific immunohistochemical staining for NSE, Syn, CgA and CK in samples from patient no. 3, a 46-year-old female, with a one-month history of backache. (A) HE, ×100 magnification; (B) HE, ×400 magnification; (C) NSE^+++^, ×200 magnification; (D) CK^++^, ×200 magnification; (E) CgA^+^, ×200 magnification; and (F) Syn^+^, ×200 magnification. Tumor cells with identical shape, regular arrangement and abundant cytoplasm were observed. Fine particles, and small and round nuclei were found in the middle of the cells by eosin staining. Chromatin was fine and uniform. Nuclear fission was often apparent. NSE, CK, CgA and Syn particles were located in the plasma of the tumor cells, with no expression in the intercellular substance. Computed tomography indicated that the mass was 4.5 cm^3^ and located on the tail of the pancreas. A distal pancreatectomy was performed. This patient was followed up until August 2011, with no signs of metastasis for 21 months. NSE, neuron-specific enolase; CK, cytokeratin; CgA, chromogranin A; Syn, synaptophysin.

The tumor samples were ashen and tough. In the IHC staining, the expression of CK, Syn, CgA and NSE was positive in nine (69.23%), 10 (76.92%), five (38.46%) and eight (61.54%) cases, respectively (Fig. 2).

### Median survival

**Figure 3:**
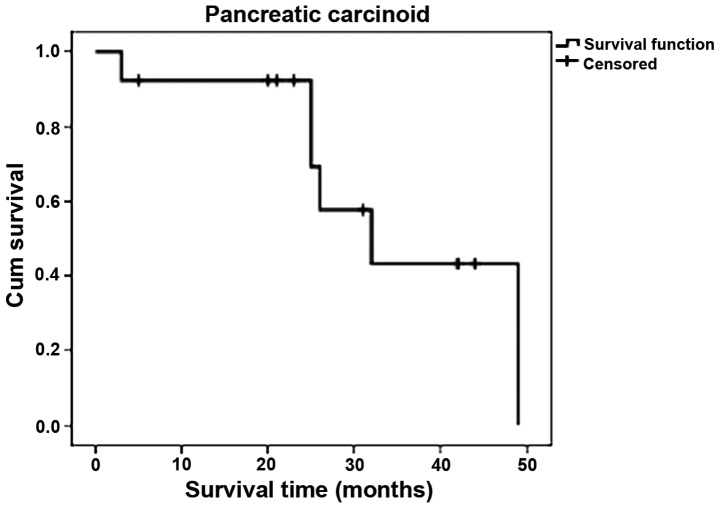
Survival curve of the 13 cases. The median survival time was 26.6 months. Seven patients are still being followed up.

The survival time of the 13 cases was calculated, and the median survival time was 26.6 months. Seven patients had been followed up until August 2011 (Fig. 3).

## Discussion

Carcinoid tumors are extremely rare malignancies that are derived from enterochromaffin cells, which come from the embryonic neural crest and are widely distributed in the digestive tract. These cells produce a large amount of polyaminoamide hormones. Carcinoid tumors originate from argentaffin cells, accounting for 0.05–0.2% of the total number of malignancies.

Carcinoid tumors have the characteristics of inhomogeneous organ distribution and mostly occur in the gastrointestinal tract; among which the appendix, small intestine and colorectum accounts for 50, 20–30 and 15%, respectively (2,3). In Tianjin Medical University Cancer Institute and Hospital, 414 patients with carcinoid tumors were treated between 1954 and May 2010, of which, 13 cases (3.14%) were pancreatic carcinoid tumors.

The occurrence of carcinoid tumors is associated with living habits, including smoking and drinking, as well as with family history, diabetes and gender (4). The tumors occur more in female than in male (5). However, in this study, there was equal incidence between female and male. Typical carcinoid syndrome presents with symptoms such as skin flushes, diarrhea, stomachaches, asthma, right-sided valvulopathy lesions and hepatomegaly. Laboratory examination show increased 5-HT content in the serum and elevated amounts of 5-HIAA in the urine. Generally, carcinoid tumors located in mid-gut secrete higher levels of 5-TH, which easily induces carcinoid syndrome. Pancreatic carcinoid tumors derived from the foregut are not typical of the syndrome. In addition, it has been reported that normal gastrointestinal tumor markers are not detected in carcinoid tumors (6). This is consistent with the present study results. Radiographic results show no specific characteristics, but heterogeneous enhancement of the arterial phase can be found on CT as a characteristic of neuroendocrine tumors. The tumors can be differentiated from pancreatic ductal adenocarcinomas. Therefore, there is cause to reject the possibility of pancreatic ductal adenocarcinoma (7). As a result, it is difficult to diagnose pancreatic carcinoid tumors prior to surgery or biopsy.

At present, the diagnosis of a pancreatic carcinoid tumor depends mainly on the pathological examination. The mucosal surface appears mostly intact and the incisal surface appeared as yellow or gray (8). Occasionally, the surface of the tumors appeared ulcerated or with hemorrhage. This appearance is similar to that of an adenocarcinoma. The tumor usually invades into the muscular layer and serosa (9). Numerous patients develop polyphyletic carcinoid tumors. Pancreatic carcinoid tumors have similar characteristics. Typically, the cancer cells appear to be consistent in terms of morphology, i.e., they have a regular arrangement, abundant cytoplasm and a small oval nucleus located in the center. Thin and uniform chromatin, and bare karyokinesis are also their common characteristics. However, the atypical characteristics of the cells can include a polygonal or spindle shape, marked nuclear atypia, extensive chromatin and marked karyokinesis. NSE, CK, CgA and Syn can be detected by immunohistochemical staining (10) and this method can be used as an adjunct to confirm the presence of a carcinoid tumor.

Surgical resection is potentially a curative therapy for carcinoid tumors. The choice of surgical methods depends on tumor size, position, infiltration and lymph node metastasis, with tumor size and infiltration being the major contributing factors (11). However, owing to the unique position of the pancreas and the lack of typical clinical characteristics, 66–81% patients present with a large tumor size and a number with distant metastasis (12). Therefore, radical surgery cannot be performed.

Moreover, there is disagreement over the administration of somatostatin analogs to medically inoperable patients. A previous study revealed that carcinoid cells express a large number of somatostatin receptors, therefore, somatostatin analogs could be used as first-line therapy drugs for patients (13). Contrary to this, there is no evidence to confirm that somatostatin can prolong median survival time following surgery or palliative treatment. At present, surgery is the only effective treatment for carcinoid tumors due to the uncertainty on the availability of chemotherapy. Reoperation can improve the prognosis following tumor recurrence or metastasis. Therefore, an early diagnosis and surgical resection are considered as the most effective treatments for pancreatic carcinoid tumors (14,15).

Pancreatic carcinoid tumors are considered to be a low-grade malignancy that grows slowly, similar to other carcinoid tumors. The condition has a good prognosis compared to pancreatic ductal adenocarcinoma, but a worse prognosis compared with that carcinoid tumors of other organs. Previous studies have shown that the five-year survival rate of patients with pancreatic carcinoid tumors may be increased to ~35% (16,17). Patients who undergo radical surgery eventually succumb due to hepatic or intra-abdominal metastasis. In the present study, metastasis was found in three cases at the time of the primary diagnosis; these patients succumbed after 26, 32 and 49 months, respectively. Seven patients remained alive at the time of the latest follow-up in August 2011.

This present study has certain limitations. Bias may have been introduced as the follow-up was not short and due to the relatively small number of patients included in the study. A larger study group is required to confirm these findings. The follow-up of the remaining patients will be continued.

In conclusion, pancreatic carcinoid has no typical syndrome and only the pathological results are able to confirm the diagnosis. Chemotherapy may not have a significant effect on the treatment of pancreatic carcinoid tumors, while surgery is the most effective treatment. Surgery for the reduction of the primary tumor and metastasis result in a appreciable improvement in the survival time and quality of life.

## Figures and Tables

**Table I:** Clinical characteristics and prognosis of patients.

Patient no.	Year admitted	Age, years	Gender	Symptoms	Tumor marker	Location and size, cm	Surgery	Treatment	Specific staining	Survival, months
1	2012	64	Male	Upper abdominal pain	Negative	Head: 6×8×4 and homotype clumping in portal vein	PD	None	NSE^+^, CK^−^, CgA^−^, Syn^−^ NSE^+^, CK^−^, CgA^−^, Syn^−^	49
2	2002	47	Male	Jaundice	Negative	Head: 8×10	Choledochojejunostomy	None		25
3	2000	46	Female	Osphyalgia	Negative	Body and tail: 4.5×4.5	Splenectomy and DP	None	NSE^+^, CK^−^, CgA^−^, Syn^+^	44+
4	2009	46	Male	Tumor detected upon physical examination	Ferritin, 240.8 μg/l	Head: 5	PD and lymphadenectomy	None	NSE^−^, CK^−^, CgA^−^, Syn^+^	42+
5	2008	44	Male	Nausea, backache and loss of weight	Negative	Head: 1	Local excision	Cisplatin plus docetaxel and biotherapy	NSE^++^, CK^+^, CgA^+^, Syn^+^	23+
6	2009	39	Male	Upper abdominal pain	Negative	Head: 6.7×5.2 and bolt in superior mesenteric venous	PD and thrombectomy from superior mesenteric venous	0.9 mg/day octreotide and directional radiation	NSE^+^, CK^+^, CgA^+^, Syn^+^	20+
7	2009	53	Male	Tumor detected upon physical examination	CEA, 5.25 μg/l	Tail: 8.5×7.9	Splenectomy and DP	None	NSE^−^, CK^+^, CgA^−^, Syn^+^	31+
8	2008	41	Female	Full distention and accentuation after taking food	Negative	Head: 6.8×5.2	Total pancreatectomy	Octreotide	NSE^+++^, CK^++^, CgA^−^, Syn^+^	25
9	2009	64	Female	Upper abdominal pain	Negative	Body and tail: 7×5	Splenectomy and DP	None	NSE^−^, CK^+^, CgA^++^, Syn^+^	21+
10	2010	47	Male	Headache	Negative	Tail: 6.2×7.8	Splenectomy and DP	Octreotide	NSE^−^, CK^+^, CgA^−^, Syn^++^	32
11	2009	62	Male	Diarrhea	Negative	Head: 6.5×7.0 and liver metastasis	PD	None	NSE^−^, CK^+^, CgA^−^, Syn^+^	26
12	2011	60	Male	Upper abdominal pain	Negative	Body and tail: 10×10	Splenectomy and DP	Octreotide acetate	NSE^+^, CK^+^, CgA^+^, Syn^+^	3
13	2005	42	Male	Upper abdominal pain and loss of weight	Negative	Head: 10 and invasion of SMV, distal common bile duct, duodenum	Choledochojejunostomy and gastrojejunostomy	Cisplatin plus docetaxel	NSE^++^, CK^+^, CgA^+^, Syn^−^	5+

Staining intensity was scored according to the percentage of positively stained cells as follows: −, <5%: +, ≥5 but <25%; ++, 25–50%; and +++, >50%. PD, pancreatoduodenectomy; DP, distal pancreatectomy; SMV, superior mesenteric vein; NSE, neuron-specific enolase; CK, cytokeratin; CgA, chromogranin A; Syn, synaptophysin; CEA, carcinoembryonic antigen.
